# Complete Genome Sequences of *Caldicellulosiruptor* sp. Strain Rt8.B8, *Caldicellulosiruptor* sp. Strain Wai35.B1, and “*Thermoanaerobacter cellulolyticus*”

**DOI:** 10.1128/genomeA.00440-15

**Published:** 2015-05-14

**Authors:** Laura L. Lee, Javier A. Izquierdo, Sara E. Blumer-Schuette, Jeffrey V. Zurawski, Jonathan M. Conway, Robert W. Cottingham, Marcel Huntemann, Alex Copeland, I-Min A. Chen, Nikos Kyrpides, Victor Markowitz, Krishnaveni Palaniappan, Natalia Ivanova, Natalia Mikhailova, Galina Ovchinnikova, Evan Andersen, Amrita Pati, Dimitrios Stamatis, T.B.K. Reddy, Nicole Shapiro, Henrik P. Nordberg, Michael N. Cantor, Susan X. Hua, Tanja Woyke, Robert M. Kelly

**Affiliations:** a Department of Chemical and Biomolecular Engineering, North Carolina State University, Raleigh, North Carolina, USA; b BioEnergy Science Center and Biosciences Division, Oak Ridge National Laboratory, Oak Ridge, Tennessee, USA; c DOE Joint Genome Institute, Walnut Creek, California, USA

## Abstract

The genus *Caldicellulosiruptor* contains extremely thermophilic, cellulolytic bacteria capable of lignocellulose deconstruction. Currently, complete genome sequences for eleven *Caldicellulosiruptor* species are available. Here, we report genome sequences for three additional *Caldicellulosiruptor* species: Rt8.B8 DSM 8990 (New Zealand), Wai35.B1 DSM 8977 (New Zealand), and “*Thermoanaerobacter cellulolyticus*” strain NA10 DSM 8991 (Japan).

## GENOME ANNOUNCEMENT

Lignocellulose-degrading microorganisms are of considerable interest for their use in the production of fuels and chemicals from renewable feedstocks. The genus *Caldicellulosiruptor* contains the most thermophilic, plant biomass–degrading bacteria isolated thus far, with optimal growth temperatures between 70 and 78°C (1). To date, complete genome sequences are available for 11 *Caldicellulosiruptor* species (1–7). From this information, it was determined that the *Caldicellulosiruptor* pan genome is still open, indicating that there is additional diversity within the genus, potentially involving yet-to-be discovered biomass-degrading enzymes and pathways. In light of this, three *Caldicellulosiruptor* isolates previously isolated from terrestrial hot springs were genome sequenced. Two were isolated from sites in New Zealand: *Caldicellulosiruptor* sp. strain Rt8.B8 DSM 8990 and strain Wai35.B1 DSM 8977 from Rotorua and Waimangu, respectively (8). The third, from Nozawa Hot Spring, Nagano Prefecture, Japan, was originally classified as “*Thermoanaerobacter cellulolyticus*” strain NA10 DSM 8991 (9, 10), although its genome sequence indicates that it belongs to the genus *Caldicellulosiruptor*.

The draft genomes of *T. cellulolyticus* NA10, *Caldicellulosiruptor* sp. strain Rt8.B8, and *Caldicellulosiruptor* sp. strain Wai35.B1 were produced by constructing Pacific Biosciences (PacBio) SMRTbell libraries, sequencing on the PacBio RS platform (11), and correcting errors on the Illumina platform for each microorganism at the DOE Joint Genome Institute (JGI). This generated 81,181, 150,202, and 166,964 filtered subreads totaling 349.1, 591.3, and 670.4 Mbp for *T. cellulolyticus* NA10, *Caldicellulosiruptor* sp. strain Rt8.B8, and *Caldicellulosiruptor* sp. strain Wai35.B1, respectively. All raw reads were accumulated via HGAP (12) and classified into genes using Prodigal (13) along with GenePRIMP (14). The predicted coding regions were then checked against the National Center for Biotechnology Information (NCBI), UniProt, TIGRFam, Pfam, KEGG, COG, and InterPro databases to annotate the genomes within the Integrated Microbial Genomes (IMG) platform (http://img.jgi.doe.gov). Specific genes, such as tRNAs, rRNAs, and other noncoding RNAs, were identified by searching the genome with the tRNAScanSE tool (15), SILVA rRNA gene models (16), and INFERNAL (http://infernal.janelia.org).

The final draft assembly of *T. cellulolyticus* NA10 DSM 8991 contained 12 contigs in 12 scaffolds, totaling 2,514,985 bp, with an input read coverage of 88.9×. The final draft assembly of *Caldicellulosiruptor* sp. strain Rt8.B8 contained 2 contigs in 2 scaffolds, totaling 2,488,483 bp in size, with an input read coverage of 165.6×. Lastly, the final draft assembly of *Caldicellulosiruptor* sp. strain Wai35.B1 contained 1 contig in 1 scaffold, totaling 2,834,482 bp in size, with an input read coverage of 175.6×. The G+C content was 35.39%, 36.49%, and 35.78% for *T. cellulolyticus* NA10, *Caldicellulosiruptor* sp. strain Rt8.B8, and *Caldicellulosiruptor* sp. strain Wai35.B1, respectively. We expect that the novel features we are identifying in these genomes will further contribute to our understanding of the metabolic diversity of lignocellulolytic capabilities within the *Caldicellulosiruptor* genus.

### Nucleotide sequence accession numbers. 

These whole-genome shotgun projects have been deposited at DDBJ/EMBL/GenBank under the accession numbers LACN00000000, LACO00000000, and LACM00000000 for *T. cellulolyticus* NA10 DSM 8991, *Caldicellulosiruptor* sp. strain Rt8.B8 DSM 8990, and *Caldicellulosiruptor* sp. strain Wai35.B1 DSM 8977, respectively. The versions described in this paper are versions LACN01000000, LACO01000000, and LACM01000000.
